# Deep proteome profiling promotes whole proteome characterization and drug discovery for esophageal squamous cell carcinoma

**DOI:** 10.20892/j.issn.2095-3941.2022.0024

**Published:** 2022-03-14

**Authors:** Wei Liu, Yongping Cui, Wen Liu, Zhihua Liu, Liyan Xu, Enmin Li

**Affiliations:** 1 College of Science, Heilongjiang Institute of Technology, Harbin 150050, China; 2 Guangdong Provincial Key Laboratory of Infectious Diseases and Molecular Immunopathology, the Key Laboratory of Molecular Biology for High Cancer Incidence Coastal Chaoshan Area, Shantou University Medical College, Shantou 515041, China; 3 Department of Pathology, Key Laboratory of Cellular Physiology (Shanxi Medical University), Ministry of Education, Taiyuan 030001, China; 4 Fujian Provincial Key Laboratory of Innovative Drug Target Research, School of Pharmaceutical Sciences, Xiamen University, Xiamen 361005, China; 5 State Key Lab of Molecular Oncology, National Cancer Center/Cancer Hospital, Chinese Academy of Medical Sciences and Peking Union Medical College, Beijing 100021, China

Proteins are key players in various cellular processes. As the ultimate executors of cellular processes, they play essential roles in linking genotypes to phenotypes. Abnormalities in protein expression and post-translational modifications (PTMs) are closely associated with the initiation and development of cancer, and they carry biological information inaccessible to genomics and transcriptomics^1^. Proteomics complements genomic and transcriptomic data, thus enabling comprehensive analysis of cancer pathogenesis and accelerating biomedical research^2^. Owing to the limitations of protein quantification instrumentation and methods, the development of proteomics has substantially lagged behind that of genomics and transcriptomics. Fortunately, recent technological advances in mass spectrometry-based proteomics have substantially increased the number of proteins that can be quantified (at high resolution) and the number of samples that can be profiled within a reasonable time frame (high throughput)^3^. Consequently, whole proteome quantification can now be performed on a large scale, thereby enabling proteome characterization with greater statistical significance, identification of proteomic subtypes, and discovery of potential drugs. We refer to the high-resolution, high-throughput proteomics profiling of large-scale samples as deep proteome profiling.

For the human cancer proteome, the National Cancer Institute’s Clinical Proteomic Tumor Analysis Consortium (CPTAC) (USA), launched in 2011, has performed deep proteome profiling of more than 10 types of tumors, such as colorectal cancer, breast cancer, ovarian tumors, and glioblastoma (**Figure 1**). Other groups have also reported deep proteome profiling studies on various types of cancers, such as hepatocellular carcinoma, gastric cancer, and lung cancer (**Figure 1**).

**Figure 1:**
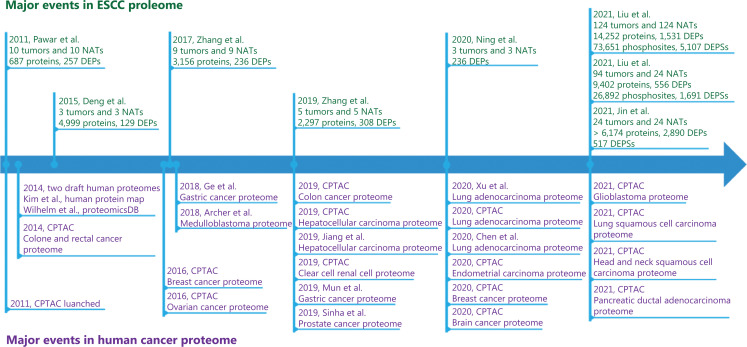
Major events in ESCC proteomic research and human cancer proteomics. CPTAC: Clinical Proteomic Tumor Analysis Consortium; NAT: normal adjacent tissues; DEP: differentially expressed protein; DEPS: differentially expressed phosphorylation sites.

For esophageal squamous cell carcinoma (ESCC), one of the most aggressive cancer types and the fourth leading cause of cancer-related deaths in China, several proteomic studies in the past decade have characterized the ESCC proteome, and identified protein biological markers and drug targets (**Figure 1**)^4–8^. However, these studies have been limited by the small numbers of samples (≤ 20) and detected proteins (< 5,000). Recently, the Li group at Shantou University Medical College and the Liu group have reported large-scale deep proteome profiling of patients with ESCC^9,10^, thus enabling the characterization of the whole proteome of ESCC, and revealing potential drugs and therapeutic targets for ESCC treatment at the proteomic level.

## Deep proteome profiling promotes whole proteome characterization of ESCC

The Li group has analyzed 124 paired tumor and non-tumor tissues from patients with ESCC by using isobaric tandem mass tag-based proteomics^9^, and has identified a total of 14,252 proteins. The Liu group has analyzed 94 tumor tissues and 24 non-tumor esophageal tissues by using isobaric tags for relative and absolute quantification (iTRAQ), and has identified a total of 9,402 proteins^10^, 9,135 (97.2%) of which were identified in Li’s study. Most proteins identified in Li’s study were distributed in the nucleus, plasma membrane, cytosol, mitochondria, and extracellular space. A total of 7,265 proteins were significantly dysregulated. Of these, 1,531 proteins exhibited a fold-change greater than 1.5, and 784 and 747 proteins were upregulated and downregulated, respectively, in tumors compared with paired non-tumors. The proteins with altered abundance in tumors were exceptionally enriched in extracellular proteins, in agreement with the results of Liu’s study, thereby suggesting that the tumor microenvironment undergoes drastic changes during tumorigenesis. Most of the esophagus-specific proteins, such as KRT4, KLK13, KRT78, SPINK5, SPINK7, and CRNN, were significantly downregulated in tumors, thus indicating that loss of esophageal identity is a characteristic of ESCC.

Functional enrichment analysis indicated that ESCC is characterized mainly by elevated levels of proteins involved in epidermal mesenchymal transition, DNA replication, cell cycle, ECM-receptor interaction, focal adhesion, and immune response pathways, and by diminished levels of proteins involved in tight junctions, adherens junctions, metabolism, and estrogen response-related pathways. Notably, cell cycle proteins were also up-regulated, but the proteins involved in ECM-receptor interaction and focal adhesion pathways were down-regulated in Liu’s cohort. Therefore, studies on the population heterogeneity of ESCC and multi-center comparative proteomics studies of ESCC are needed in the future.

Proteomics analysis confirmed several of the frequently altered genes and pathways in ESCC revealed by genomic analysis, such as CCND1, CDK4, and CKD6 in cell cycle, and EGFR in the RTK/PI3K signaling pathway. The upregulation of these proteins was consistent with their observed amplification in tumor samples. Meanwhile, several inconsistent changes between the genome and the proteome were observed. Some genes with frequent genomic mutations (SNV/indel) or deletions, such as CDKN2A, RB1, and TP53, exhibited higher protein abundance in tumor samples. In contrast, several genes that are frequently mutated or amplified, such as KRAS, AKT1, and PIK3CA in the RTK/PI3K signaling pathway, were found to exhibit no changes at the protein level. In addition, many metastasis and immune response proteins (such as MMP1, MMP2, MMP3, MMP9, MX1, IDO1, IFIT1, and IFIT3) and epigenetic regulators (such as the histone methyltransferase and demethylases KDM1A, KDM3A, KDM3B, KDM5C, ASH1L, NSD2, and NSD3; histone acetyltransferase EP300 and CREBBP; and chromatin structure modifier ARID1A, SMARCC1, and SMARCC2) were found to be upregulated in tumors, without showing genomic alterations or frequent inactivating mutations. These connections and discordances between proteomic and genomic alterations provide valuable clues for deciphering the pathogenesis of ESCC.

In addition, both studies have characterized the phosphoproteomics of ESCC. Li performed label-free phosphoproteomics on 31 pairs of tumor and non-tumor esophageal tissues, and detected 73,651 phosphosites in 7,943 proteins. Of the 26,892 phosphorylation sites detected in Liu’s study^10^, 19,850 (73.8%) were also identified in Li’s study. Functional enrichment analysis revealed that proteins in the cell cycle and spliceosome pathway are often highly phosphorylated, in agreement with Liu’s findings. Spliceosome proteins including SRSF1, SRSF2, SRSF7, SRSF8, and SRSF10 were found to be hyperphosphorylated. PTM-SEA analysis revealed that the activity of kinases including CDK1, CDK2, CDC7, CHEK2, MELK, CDK7, IKK, PIM1, and CK2A1 was upregulated in tumors compared with non-tumor samples. These kinases may serve as potential druggable targets for ESCC.

## Deep proteome profiling promotes drug discovery for ESCC

Large-scale deep proteome profiling of patients with ESCC has allowed the 2 studies to identify clinically relevant proteomic subtypes of ESCC and to predict potential drugs specific to aggressive subtypes. Li’s study identified 2 clinically relevant proteomic subtypes, S1 and S2, on the basis of consensus clustering of protein expression data^9^. Patients with the S2 subtype had poorer overall survival and disease-free survival outcomes than those with the S1 subtype. Liu’s study defined 3 major proteomic subtypes (S-I, S-II, and S-III) in their tumor cohort of ESCC, among which the S-III subtype was more aggressive^10^. The elevated proteomic and phosphoproteomic levels in spliceosome pathways in the aggressive subtype in both cohorts confirmed that the activity of this pathway leads to a malignant phenotype.

According to the differentially expressed proteins between the S1 and S2 subtypes, 6 candidate drugs were predicted for the treatment of patients with the S2 subtype in Li’s study^9^. Among them, 3 candidate drugs—GW8510, menadione, and sulconazole—were validated to significantly inhibit the proliferation of ESCC cell lines and xenografts. To investigate the inhibitory mechanisms of GW8510, menadione, and sulconazole against ESCC, Li’s study quantified the proteomes of ESCC cells under the 3 drug treatments, and verified that all 3 drugs inhibited ESCC cell proliferation by down-regulating the up-regulated proteins in the S2 subtype and activating the down-regulated proteins in the S2 subtype^9^. Liu’s study focused on kinases and phosphatases with dysregulated activity in patients with the S-III subtype, and identified 2 highly expressed protein phosphatase 1 inhibitors, CD2BP2 and WBP11, which may function as enhancers of CLK1 kinase and play key roles in ESCC cell proliferation^10^. Furthermore, a CLK1 inhibitor, TG003, was validated to decrease tumor growth in xenografts with high expression of CD2BP2 and WBP11.

In addition, Li’s study identified 110 proteins that were significantly dysregulated in S2 subtype and significantly correlated with overall survival outcomes. Most of these were plasma proteins, transmembrane proteins, membrane proteins, drug targets (DrugBank), enzymes, CD markers, transporters, or transcription factors, which are enriched with druggable targets. Similarly, Jin et al.^11^ identified 32 potential cancer drivers and 29 known kinases with more than four-fold higher expression in ESCC than non-ESCC tissues. Among them, 7 cancer drivers and 25 kinases have targeted inhibitors in DrugBank and PKIDB, which could be tested as candidate therapeutics for ESCC. These altered cancer drivers and kinases revealed by deep proteomic data may give rise to new therapeutic targets.

## Perspectives

The studies discussed above have characterized the whole ESCC proteome, thus providing valuable resources for deciphering the pathogenesis of ESCC. Nevertheless, several issues remain to be addressed. First, the in-depth elucidation of the pathogenic mechanism of ESCC requires the integration of multiple omics data, such as that from genomics, transcriptomics, and proteomics, for joint analysis. Large-scale proteogenomics analysis has been performed on a variety of cancer types, such as colorectal cancer, gastric cancer, hepatocellular carcinoma, and lung adenocarcinoma. Although Li’s study integrated proteomic data with previously published genomic data, the proteomic and genome data were not from the same samples, thus hindering in-depth analysis of the pathogenic mechanisms. Large-scale and parallel multi-omics data measurements and integrated analysis based on the same samples with ESCC are needed.

Second, the activities and functions of proteins are tightly regulated by PTMs. Among the more than 200 types of PTMs^12^, the most common include phosphorylation, ubiquitination, acetylation, and glycosylation. The 2 studies discussed above have characterized the phosphoproteome of ESCC^9,10^. Potential phosphoproteome-driven drugs (such as TG003) and therapeutic targets, particularly kinases, have been identified. However, large-scale detection, quantification, and proteomic analysis of other common PTMs in ESCC, such as ubiquitination and acetylation, are needed.

Third, most of the proteomic data in existing studies were quantified in esophageal tissues. In clinical applications, protein biomarkers that can be assessed non-invasively often have greater practical value. Therefore, characterization of the body fluids, serum, or plasma proteomics of patients with ESCC is therefore necessary.

ESCC shows a remarkable variation in incidence globally. Genomic analysis of patients with ESCC from 8 countries with varying incidence rates has indicated similar mutational profiles of ESCC across all countries studied^13^. However, at the proteomic level, Li’s study^9^ and Liu’s study^10^ on patients with ESCC from 2 regions of China have revealed several inconsistent results. Different proteomic subtypes of ESCC were defined separately in each study. Although some of the same characteristics were shared by more aggressive subtypes, integration of ESCC samples from multiple centers or multiple countries to define more generalized proteomic subtypes is required to improve ESCC stratification and drug development.

Finally, drugs exert their effects primarily through direct interaction with proteins. Quantitative proteomics has inherent advantages in elucidating the mechanisms of drug action, and facilitating drug discovery and development. Deep proteomic data have shown higher utility than genomic or transcriptomic data in predicting both drug-target interactions and drug sensitivity^14^. The above potential therapeutic targets and drugs revealed through proteomic analysis provide further opportunities for effective ESCC treatment strategies eliciting high therapeutic responses. Further screening of candidate drugs, verifying their efficacy, and promoting the translation of drugs into clinical practice will be a driving force to improve the clinical treatment of ESCC.
